# PXDN as a pan-cancer biomarker and promotes tumor progress via immune inhibition in nasopharyngeal carcinoma

**DOI:** 10.3389/fonc.2024.1463011

**Published:** 2024-09-27

**Authors:** Yu-Chun Li, Yan-Hong Xiao, Fei-Xiong Chen, Si-Yu Xiao, Jian-Mei Lin, Shu-Tao Cai, Cui-Lan Zeng, Xue-Yan Ye, Xu-Fa Yu, Li Yuan, Shi-Bing Li

**Affiliations:** ^1^ Department of Clinical Laboratory, The Sixth Affiliated Hospital, Sun Yat-sen University, Guangzhou, China; ^2^ Biomedical Innovation Center, The Sixth Affiliated Hospital, Sun Yat-sen University, Guangzhou, China; ^3^ State Key Laboratory of Oncology in South China, Collaborative Innovation Center for Cancer Medicine, Guangdong Key Laboratory of Nasopharyngeal Carcinoma Diagnosis and Therapy, Sun Yat-sen University Cancer Center, Sun Yat-sen University, Guangzhou, China

**Keywords:** PXDN, tumor progress, nasopharyngeal carcinoma, pan-cancer biomarker, tumor micro environment, immune inhibition

## Abstract

**Background:**

Investigating oncogenes and the mechanisms driving oncogenic processes in human tumors is imperative for the development of efficient therapies. Peroxidasin (PXDN) has been reported to play a critical role in tissue development and homeostasis. However, the role of PXDN in the occurrence and development of Nasopharyngeal Carcinoma (NPC) remains unknown.

**Methods:**

Data from multiple databases, including GEO and TCGA, were used to analyze the expression levels of PXDN. Taking nasopharyngeal carcinoma as an example, *in vitro* experiments were conducted to explore the biological functions of PXDN. Overexpression of stable cell lines was achieved through lentiviral infection, cell proliferation was examined using CCK8 and BrdU incorporation assays, and clone formation experiments were performed to assess cell growth. Transwell and wound healing assays were employed to evaluate cell invasion and migration abilities. Additionally, immunofluorescence staining with multiple targets was used to analyze the immune microenvironment of the tumor tissues. Co-culture experiments, followed by clone formation and CFSE incorporation assays, were conducted to observe the impact of NPC stable cell lines on T cells. Flow cytometry was performed to detect surface markers and cytokines in T cells after co-culture to assess T cell function.

**Results:**

PXDN was highly expressed in multiple tumors, and its high expression and mutation profile were correlated with poor survival. Functionally, PXDN plays a crucial role in promoting oncogenic processes by enhancing NPC cell proliferation and metastasis. Mechanistically, PXDN activates extracellular matrix (ECM) signaling pathways while simultaneously inhibiting T-cell infiltration and activation, thereby facilitating cancer progression.

**Conclusion:**

We characterized PXDN as a valuable biomarker for pan-cancer diagnosis and prognosis. We also uncovered new oncogenic roles for PXDN in promoting cancer progression and regulating T-cell immunosuppressive function in NPC.

## Background

Peroxidasin (PXDN), also known as vascular peroxidase 1 (VPO1), is a member of the heme-containing peroxidase family (1). This family of proteins is highly expressed in cardiovascular tissues and plays an important role in cardiovascular diseases (2). PXDN is also upregulated in aortas of rats with type 2 diabetes (3). In the past decade, research on PXDN has revealed its diverse physiological roles as a multi-functional peroxidase, including functions in host defense, atherosclerosis, heart and vessel remodeling, endothelial dysfunction, and fibrosis (4–6). Increasing evidence shows that PXDN induces endothelial cell death by promoting apoptosis and programmed necrosis (7, 8). PXDN deficiency can increase ROS levels in hepatocytes, leading to the activation of hypoxic signals and causing the recruitment and reprogramming of macrophages, which in turn ameliorates hepatocyte fibrosis (6, 9). Recently, many studies have found that PXDN plays an important role in tumor tumorigenesis and development, such as in oral cancer (10) and ovarian cancer (11). However, there are no relevant research reports on the involvement of PXDN in the occurrence and development of nasopharyngeal carcinoma (NPC).

Nasopharyngeal carcinoma (NPC) is a malignant tumor of the top and lateral walls of the nasopharyngeal cavity. Its incidence shows obvious familial aggregation and regional characteristics (12). It is most common in southeastern Asia and parts of northern Africa, and especially in southern China. At the time of initial diagnosis, over 60% of patients are already in an advanced stage of cancer, leading to poor treatment prognosis (13). Epstein–Barr virus (EBV) infection, alterations in oncogenes or tumor suppressor genes, epigenetic changes, environmental carcinogens, and immune escape are involved in the occurrence and development of NPC (14, 15). EBV infection is the most important pathogenic factor in NPC (16). Meanwhile, the abnormal expression of oncogenes not only causes abnormal biological functions in tumor cells but also has a crucial impact on the function of immune cells (such as T cells) in the tumor microenvironment, which is reflected in the exhaustion of T cells in the tumor microenvironment (17). However, oncogenes that promote tumor progression by affecting T cell function in the tumor microenvironment need to be further explored. As a highly lymphocyte-infiltrating tumor, NPC is highly associated with the host immune system. In the NPC microenvironment, although there is a large amount of T-cell infiltration, T cells are often in a state of immune exhaustion, resulting in the immune escape of tumor cells (18). Exploring T-cell exhaustion during the development of NPC will help clarify the pathogenesis and provide new targets for targeted therapy of NPC.

In this study, we investigated the expression of PXDN in various cancers and found that it is highly expressed and serves as a pan-cancer biomarker. In addition, we assessed the correlation between PXDN and survival status, mutation status, and relevant cellular pathways. Comprehensive analysis revealed that upregulation of PXDN is associated with worse clinical prognosis in various human cancers. We explored the biological function of PXDN in NPC and found that PXDN overexpression promoted cancer cell proliferation and invasion. We also assessed the relationship between PXDN expression and T-cell infiltration in the tumor microenvironment, which revealed that high PXDN expression inhibited T-cell anti-tumor immunity in NPC.

## Materials and methods

### Tissue samples

NPC specimens were obtained from histologically and clinically diagnosed NPC patients at the Sun Yat-sen University Cancer Center. PBMCs were isolated from the blood samples of healthy donors. This study was approved by the Institutional Research Ethics Committee of the Sixth Affiliated Hospital of Sun Yat-sen University (2023ZSLYEC-629). Informed consent was obtained from all subjects, and the experiments conformed to the principles of the WMA Declaration of Helsinki and the Department of Health and Human Services Belmont Report.

### Gene expression analysis

The expression levels of PXDN in tumor types and adjacent normal tissues were analyzed using TIMER2 (https://cistrome.shinyapps.io/timer/), GEPIA (http://gepia.cancer-pku.cn/detail.php), and UALCAN (http://ualcan.path.uab.edu/analysis-prot.html). The overall survival and disease-free survival significance map data and survival plots of PXDN were obtained from GEPIA. The cBioPortal tool (https://www.cbioportal.org/) was used to collect data on alteration frequency, mutation type, mutated site information, copy number alteration (CAN), and three-dimensional (3D) protein structure across all TCGA tumors. The GSE database (GSE13597 and GSE12452) was used to analyze PXDN expression and the regulated pathways in NPC. TIMER2 was used to analyze the correlation between PXDN expression and immune cell infiltration.

### Western blot

Western blot analysis was performed as previously described (19). Briefly, the indicated cells were collected and lysed by RIPA buffer on ice for 15 min. The protein concentration of the cell lysates was measured using a bicinchoninic acid (BCA) protein assay kit (Pierce). The protein bands were separated using 10% polyacrylamide SDS-PAGE, transferred to a PVDF membrane, and probed with the indicated antibodies. The blots were then incubated with species-specific HRP-conjugated secondary antibodies. Immunoreactive bands were visualized using enhanced chemiluminescence (ECL, Pierce). The following antibodies were used: anti-PXDN (Millipore, ABS1675), anti-FLAG M2 (Sigma-Aldrich, F1804), anti-FN1(CST, #63779), anti-COL1A1(CST, #39952), anti-COL3A1(CST, #30565), and anti-β-actin (Protein Tech Group Inc., 66009-1-1g). Antibodies were used at a 1:1,000 dilution for immunoblotting experiments.

### CCK8 assay

CCK8 reagent (APExBIO, K1018) was used to measure cell growth according to the manufacturer’s instructions. Briefly, 1,500 cells per well of CNE2 and 3,000 cells of HK1 were seeded in 96-well plates and cultured in RPMI1640 supplemented with 5% fetal bovine serum. Cell proliferation, reflected in OD450 nm, was examined at 1, 2, 3, 4, and 5 days following standard procedures. All experiments were repeated three times.

### BrdU incorporation assay

BrdU incorporation assay was performed as previously described (20). Briefly, cells stably over-expressing cells PXDN (6 × 10^4^) were seeded on coverslips in 24-well plates. After 24 h, the cells were incubated with BrdU (20 µM) for 2 h and stained with anti-BrdU (Abcam) according to the manufacturer’s instructions. The cells were examined under a confocal laser-scanning microscope; green indicates positive BrdU incorporation and blue staining indicates the nuclei. The following antibodies were used in this study: anti-BrdU (Abcam, ab6326) and FITC-conjugated anti-rabbit IgG (Invitrogen,Carlsbad, CA, USA).

### Colony formation assay

NPC cell lines overexpressing vector or PXDN were plated in triplicate in 6-well plates at 600 cells per well and cultured for 10 days. The cells were washed with PBS, fixed with methanol for 15 min, stained with 0.5% crystal violet for 15 min and counted. All experiments were repeated three times.

### Wound healing

NPC cell lines overexpressing vector or PXDN were plated in 6-well plates and cultured to 80% confluence. The medium was then replaced with FBS-free RPMI 1640 and the cells were cultured for 24 h. A sterile pipette tip was used to scratch and take a photo, which was taken at the same location 24 h later. The degree of wound healing was calculated, indicating cell migration ability.

### Transwell assay

NPC cell lines (5 × 10^4^ cells for the migration assay and 7.5 × 10^4^ cells for the invasion assay) with PXDN overexpression resuspended in 200 µL FBS-free RPMI 1640 were seeded in the top chamber of the transwell (for invasion assay, the chamber was coated with Matrigel). The lower chamber was filled with 500 µL of 10% FBS in RPMI 1640 as a chemoattractant. After incubation for 24 h (36 h–48 h for the invasion assay), cells on the lower surface of the membrane were fixed, stained, and counted. Each group of cells was analyzed in triplicate.

### Multiplex immunohistochemical staining

mIHC was performed as described previously (21), and sections (4 μm) from whole FFPE blocks of tumor tissues were sectioned, dewaxed, and fixed with 10% neutralized formaldehyde. Antigen retrieval was then performed for 2.5 min using heated Tris-EDTA buffer (pH 8.0 or pH 9.0). Four rounds of sequential antibody staining were performed on each section after the initial establishment of the staining conditions for each primary antibody and serial optimization. Each staining step consisted of blocking with 20% normal goat serum/fetal bovine serum in PBS and incubation with primary antibodies, followed by incubation with biotinylated anti-mouse/anti-rabbit secondary antibodies and streptavidin-horseradish peroxidase (HRP) substrate. Immunoreactive staining was then visualized using tyramide signal amplification (TSA) and the fluorophores Opal 480, 520, 570, and 690 diluted in 1× Plus Amplification Diluent. Finally, the Ab–TSA complexes were stripped in heated Tris-EDTA buffer (pH 8.0 or pH 9.0) for 2.5 min. Nuclei were counterstained with 4’,6-diamidino-2-phenylindole dihydrochloride (DAPI) and mounted using Perma Fluorfluorescence Mounting Medium (PANOVUE). The same procedure was used but without the primary antibody as a negative control. Multiplex immunohistochemical staining results were scored based on the percentage of the cell subpopulation. Computer recognition software automatically identified and counted cell subpopulations using indicator molecules. Cell quantification was performed on whole tumor sections using Halo Analysis Software (PANOVU).

### T cell culture, proliferation, and function

Peripheral blood mononuclear cells (PBMCs) were isolated using Ficoll density gradient centrifugation. T cells were isolated from the PBMSs by CD3 MicroBeads, plated in 24-well plates coated with OKT3 (1 µg/ml), and cultured with low-dose rhIL-2 (150 IU/ml) medium. The T cells were co-cultured with indicated NPC cells (20:1), the T cells were stained with 5,6-carboxyfluorescein diacetate, succinimidyl ester (CFSE; CST) to detect the T-cell proliferation by flow cytometry. For T-cell function, the surface marker TIM3 and cytokine TNFα were detected using flow cytometry.

### Statistical analysis

All statistical analyses were performed using GraphPad Prism (version 8.0; San Diego, CA, USA). Grouped data are presented as the mean ± SD or mean ± SEM, unless otherwise stated. Differences between two groups were assessed using a two-tailed unpaired t-test. Survival outcomes were compared using the Kaplan–Meier method and log-rank test. Statistical significance was set at P <0.05.

## Results

### PXDN is highly expressed in various cancers

**Figure 1:**
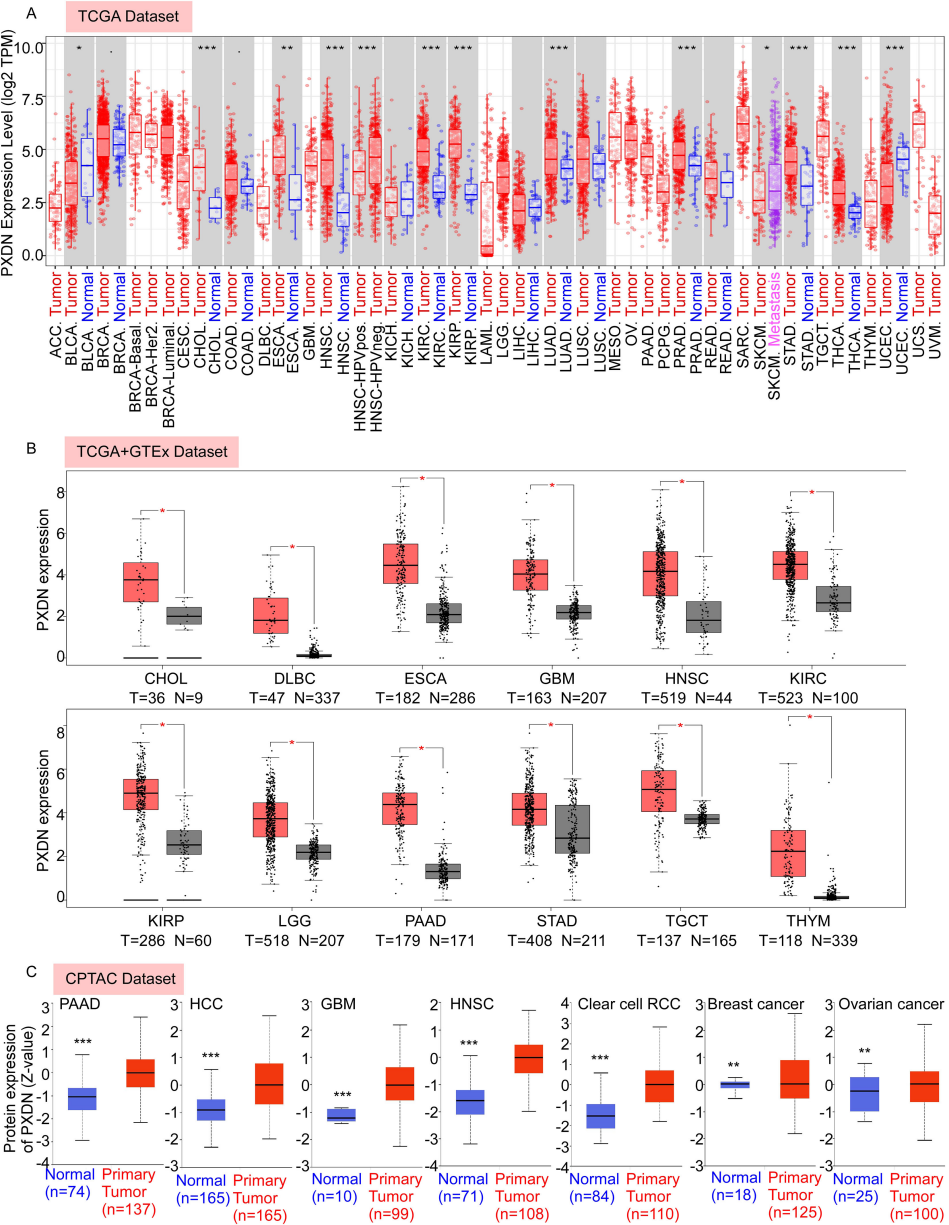
PXDN is highly expressed in various cancers. **(A)** The mRNA expression level of PXDN between tumor and adjacent normal tissues were compared in 33 types of cancers based on the TCGA database as visualized by TIMER2. **(B)** The mRNA expression level of PXDN between tumor and normal tissues were compared in CHOL, DLBC, ESCA, GBM, HNSC, KIRC, KIPP, LGG, PAAD, TCGT, and THYM based on the integrated database from TCGA and GTEx datasets as visualized by GEPIA2. **(C)** The expression level of PXDN protein between normal tissue and primary tissue were compared in PAAD, HCC, GBM, HNSC, Clear cell RCC, breast cancer, and ovarian cancer based on the CPTAC dataset. *p <0.05, **p <0.01, and ***p <0.001.

In this study, we aimed to comprehensively analyze the putative oncogenic role of human PXDN. First, we used TIMER2 to investigate the differential expression of PXDN between tumors and adjacent normal tissues for tumors represented in TCGA database. As shown in **Figure 1A**, higher expression of PXDN was observed in tumor tissues (CHOL, HNSC, KIRC, KIRP, LUAD, PRAD, STAD, THCA, ESCA, and BLCA) than in normal control tissues. In contrast, PXDN showed lower expression in the UCES tissues than in the corresponding control tissues. Remarkably, only a few tumor types (BRCA, COAD, KICH, LIHC, LUSC, and READ) showed no differential expression.

To fully explore PXDN expression in different tumors, we further assessed the differences in PXDN expression between tumor and normal tissues using the GEPIA database. We found that PXDN was highly expressed in CHOL, DLBC, ESCA, GBM, HNSC, KIRC, KIRP, LGG, PAAD, STAD, TGCT, and THYM tumor tissues (**Figure 1B**). In addition to transcription, we assessed the protein levels of PXDN using large-scale proteome data available through the National Cancer Institute’s CPTAC dataset. The results showed that the total protein expression of PXDN was significantly higher in PAAD, HCC, GBM, HNSC, Clear cell RCC, breast cancer, and ovarian cancer tissues than in normal tissues (**Figure 1C**). Overall, these results suggest that PXDN is highly expressed in most human tumors.

### High PXDN expression is correlated with poorer survival in multiple tumors

**Figure 2:**
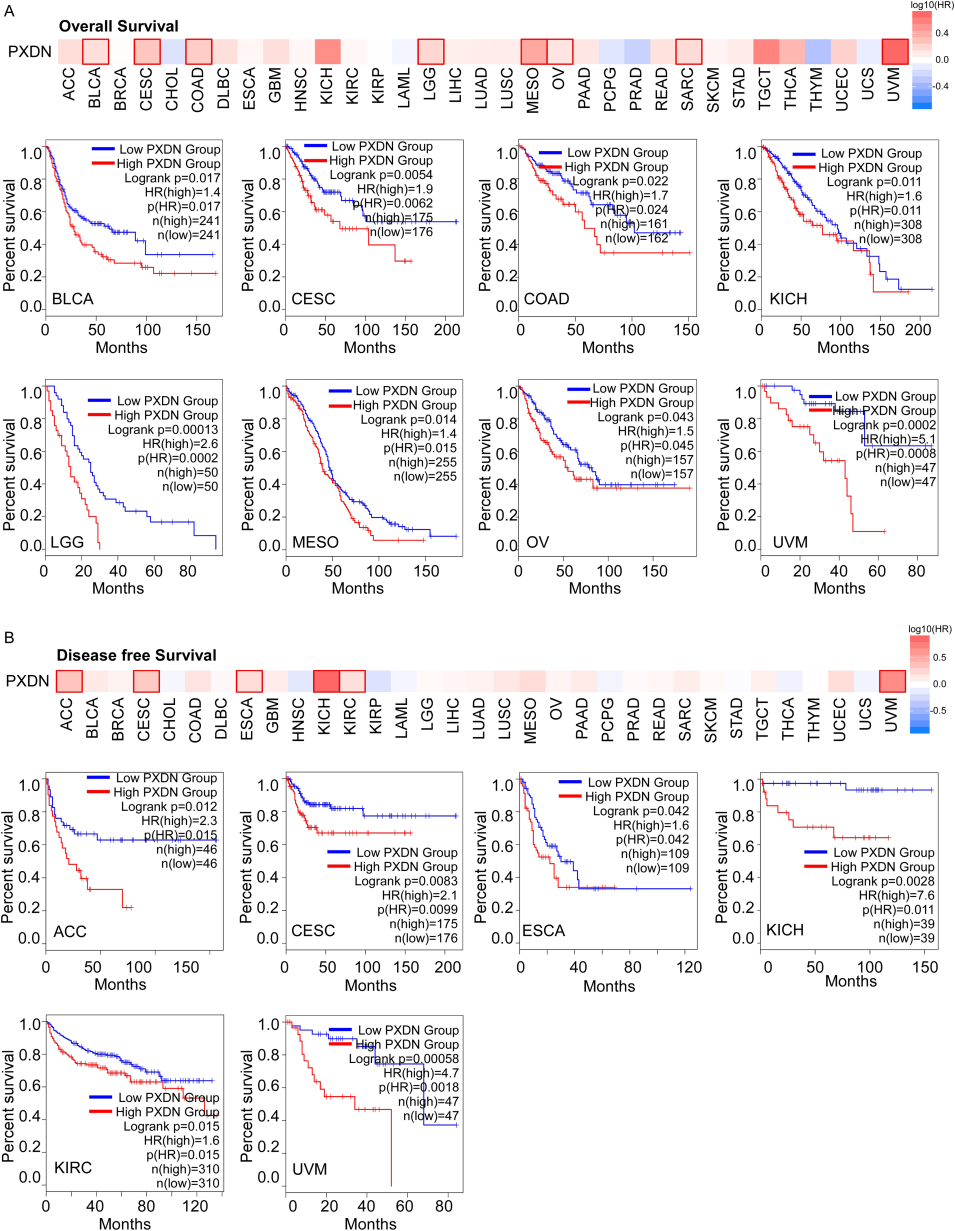
High PXDN expression is correlated with poorer survival in multiple tumors in TCGA. **(A)** Relationship between PXDN expression and overall survival in all TCGA tumors using GEPIA2. **(B)** Relationship between PXDN expression and disease-free survival in all TCGA tumors using GEPIA2. The positive results of survival map and Kaplan–Meier curves are listed.

To explore whether PXDN could be a prognostic marker in different tumors, we divided cancer cases into high- and low-expression groups according to PXDN expression levels in the TCGA dataset. We found that high expression of PXDN was associated with poor overall survival (OS) in cancers, including BLCA, CESC, COAD, KICH, LGG, MESO, OV, SARC, and UVM (**Figure 2A**). The disease-free survival (DFS) analysis showed that patients with higher PXDN expression had shorter DFS in ACC, CESC, ESCA, KICH, KIRC, and UVM (**Figure 2B**). These findings suggest that high PXDN expression is associated with poor survival prognosis in most cancers.

### The mutation feature of PXDN is correlated with clinical survival in different cancers of TCGA

**Figure 3:**
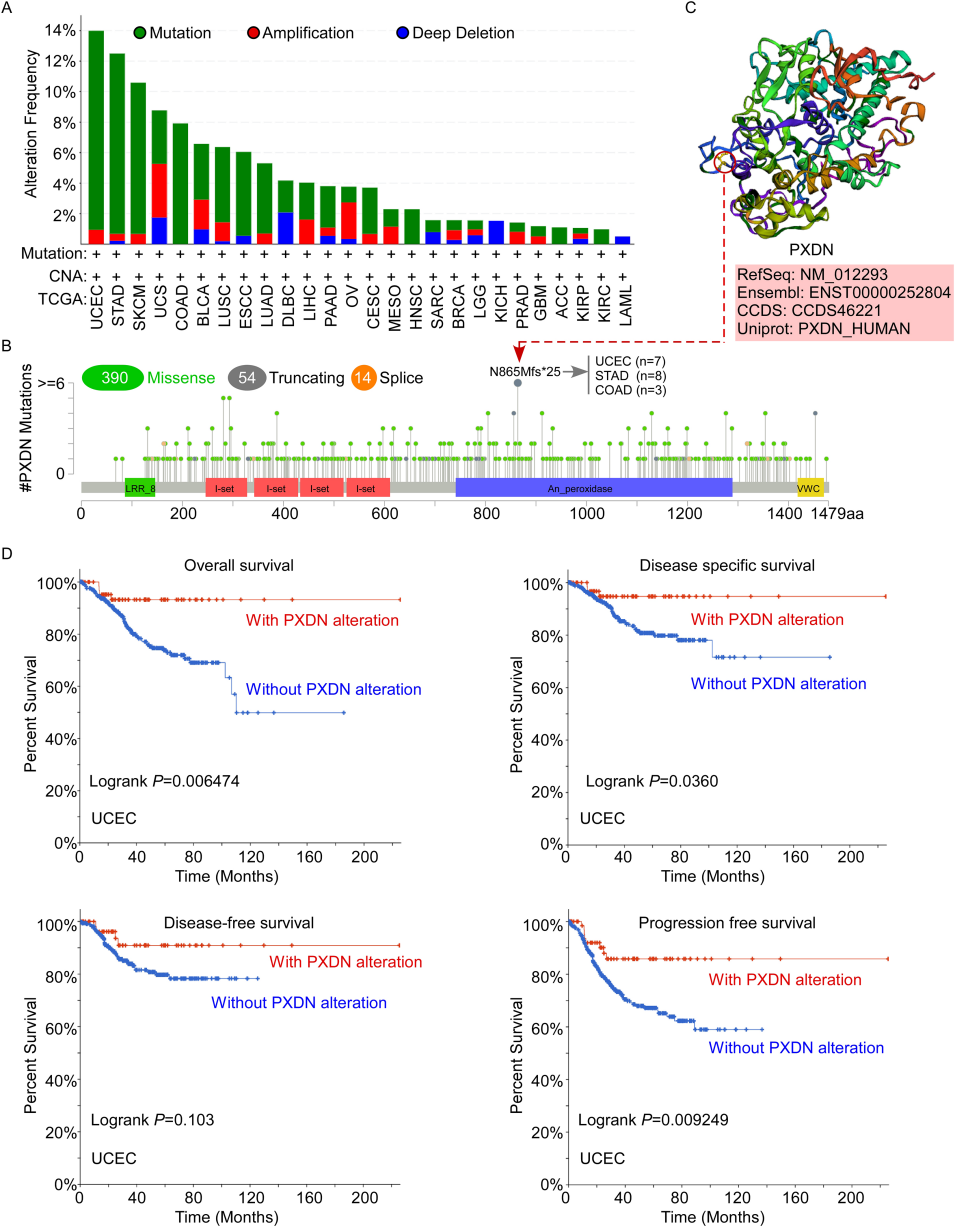
Mutation status of PXDN in TCGA tumors. **(A, B)** Mutation status of PXDN in TCGA tumors was analyzed using the cBioPortal tool and the alteration frequency with mutation types **(A)** and mutation site **(B)** are displayed. **(C)** The 3D structure of the N865 site of PXDN protein. **(D)** Analysis of the correlation between mutation status and OS, DSS, DFS, and PFS of UCEC using the cBioPortal tool.

Aberrant amplification and mutation of oncogenes have been shown to be one of the important mechanisms leading to tumorigenesis. Therefore, to better understand the tumor-promoting behavior and abnormal expression of PXDN, it is necessary to clarify the genetic alterations and copy number alterations in PXDN in different tumors. Thus, we further explored PXDN genetic alterations in human tumor samples in the cBiPortal for Cancer Genomics database. According to our analysis, the frequency of PXDN alteration (~13%) is the highest in uterine tumors with “mutation” as the primary type. UCS had the highest incidence of “amplification” type of CAN, with the frequency of ~3% (**Figure 3A**). In **Figure 3B**, we show the additional mutations and their location within PXDN and found that a missense mutation of PXDN was the main type of genetic alteration. The N865Mfs*25 alteration in the An_peroxidase domain was detected in seven cases of UCES, eight cases of STAD, and three cases of COAD. This alteration led to a frameshift mutation in the PXDN gene, changing the translation from N to M at the 865 site of the PXDN protein, ultimately resulting in truncation of the PXDN protein. We acquired the N865 site visualized in the 3D structure of the PXDN protein (**Figure 3C**). Additionally, we explored the potential association between certain genetic alterations in PXDN and clinical survival prognosis of patients with UCEC. The data indicated that UCEC patients with genetic alterations of PXDN had better OS (*p* = 0.006474), DFS (*p* = 0.036), and PFS (*p* = 0.009249), but not DFS (*p* = 0.103), compared with patients without PXDN alterations (**Figure 3D**). Overall, these results indicated that genetic alterations in PXDN are closely associated with patient survival prognosis.

### PXDN is highly expressed in NPC and positively associated with extracellular matrix organization and negatively associated with T-cell activation

**Figure 4:**
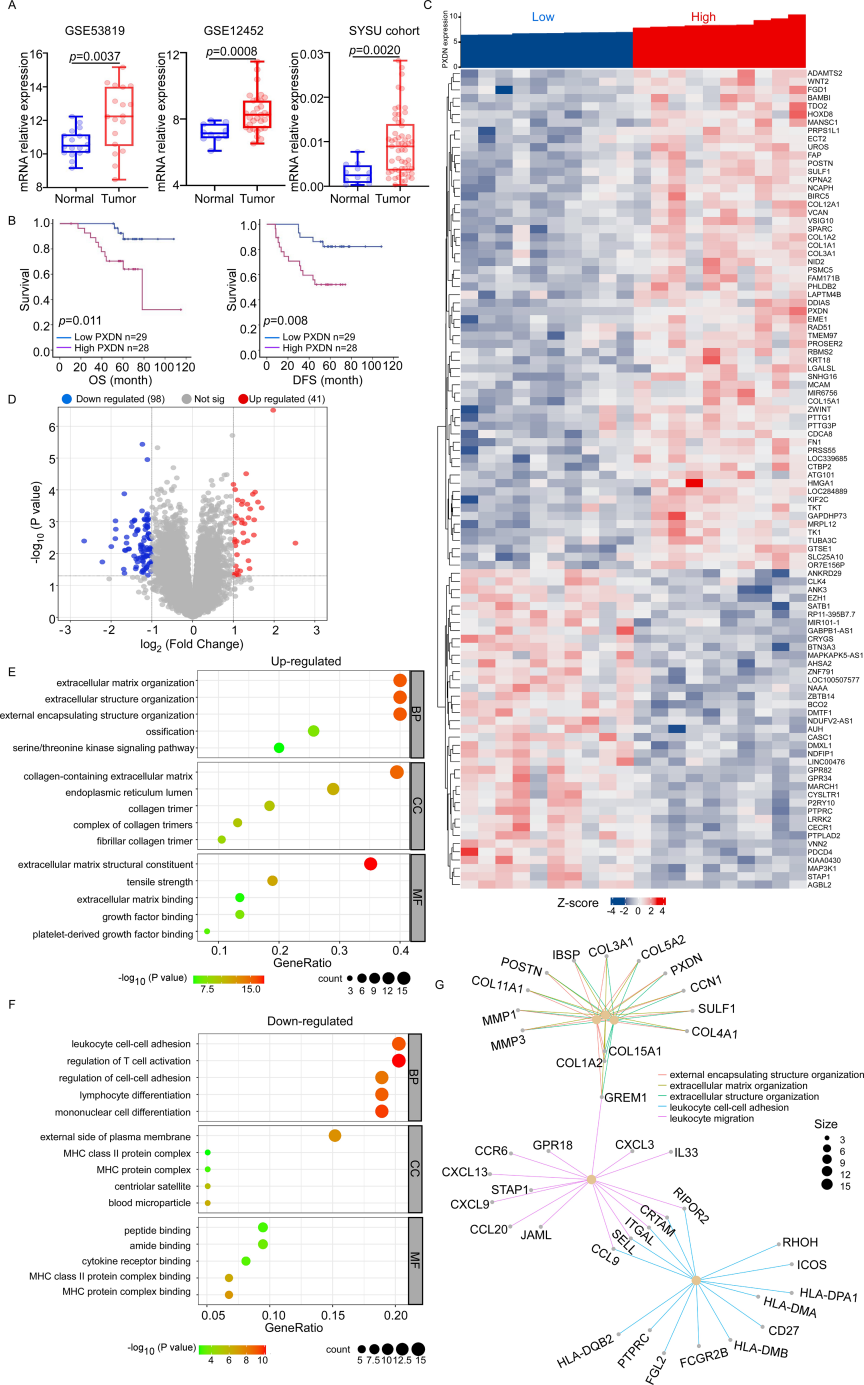
PXDN-related gene enrichment and pathway analysis in NPC. **(A)** Box plots of PXDN expression level in NPC patients and normal control. PXDN mRNA expression levels were downloaded from public data sets GSE53819 (18 cancer and 18 normal tissues) and GSE12452 (31 cancer and 10 normal tissues). We also detected PXDN mRNA expression in NPC (n = 57) and normal tissues (n = 12) from Sun-Yet Sen University Cancer Centre by q-RT-PCR. **(B)** Kaplan–Meier curves showing the overall survival (OS, left) and disease-free survival (DFS, right) curves of NPC patients with low or high PXDN expression. **(C, D)** According to PXDN expression level, the top 10 and lowest 10 samples form GSE12452 data set was selected for pathway analysis. The Heatmap **(C)** and volcano plot **(D)** showed genes upregulated or downregulated by PXDN. **(E)** The upregulated Gene Ontology term in biological process (BP), molecular function (MF) and cell component (CC) associated with PXDN. **(F)** The downregulated Gene Ontology term in biological process (BP), molecular function (MF) and cell component (CC) associated with PXDN. **(G)** The pathways affected by PXDN.

To further explore the function of PXDN in tumors, we used nasopharyngeal carcinoma as our research object because no relevant research on NPC has been reported. We performed data analysis of two public datasets for NPC (GSE53819 and GSE12452), which indicated that PXDN expression was significantly elevated in NPC samples compared to their normal counterparts (**Figure 4A**). Additionally, q-RT-PCR was used to detect PXDN mRNA expression in 57 NPC tissues and 12 normal nasopharyngeal tissues from the SYSU cohort, which also confirmed that PXDN was highly expressed in NPC tissues. The characteristics of patients with NPC are listed in **Supplementary Table 1**. Furthermore, to determine the prognostic function of PXDN in NPC, we used the Kaplan–Meier test to analyze the correlation between PXDN expression and patient survival in the SYCU cohort. Patients with high PXDN expression had shorter overall survival (OS, *P* = 0.011) and disease-free survival (DFS, *P* = 0.008) than those with low PXDN expression (**Figure 4B**). Next, we performed univariate and multivariate analyses using the Cox proportional hazards model to determine whether the PXDN mRNA expression level could serve as an independent prognostic predictor. Both univariate and multivariate analysis models revealed that the predominant independent predictors of OS and DFS were PXDN mRNA expression level and EBV DNA copy number, as shown in **Supplementary Table 2**.

The molecular functions of PXDN in NPC were further analyzed using Gene Ontology (GO) to predict PXDN-related signaling pathways in GSE12452. According to the PXDN expression level, the top 10 and lowest 10 samples from the GSE12452 dataset were selected for GO analysis. The heatmap and volcano plot showed genes upregulated or downregulated by PXDN (**Figures 4C, D**). In the upregulated pathways, the most enriched terms were “extracellular matrix organization,” “collagen-containing extracellular matrix,” and “extracellular matrix structural constituent” in biological processes (BP), cellular components (CC), and molecular functions (MF), respectively (**Figure 4E**). The downregulated pathways were associated with immune cell adhesion and activation (**Figure 4F**). In general, the pathways affected by PXDN focus on the extracellular matrix (ECM) and immune cell activation (**Figure 4G**). The EMC is reported to be an evolving structure that is produced, modified, remodeled, and maintained by the cells that reside within it. ECM pathways are often closely related to tumor metastasis. Additionally, ECM also influences the migration of immune cells that promote or prevent tumor growth, depending on the tumor type and disease stage (22). Based on these findings, PXDN is highly expressed in NPC tissues, activates the EMC pathway and inhibits immune cell activation. Therefore, we hypothesized that PXDN might affect tumor metastasis and host immunity, which requires further verification.

### PXDN promotes cell proliferation and metastasis

**Figure 5:**
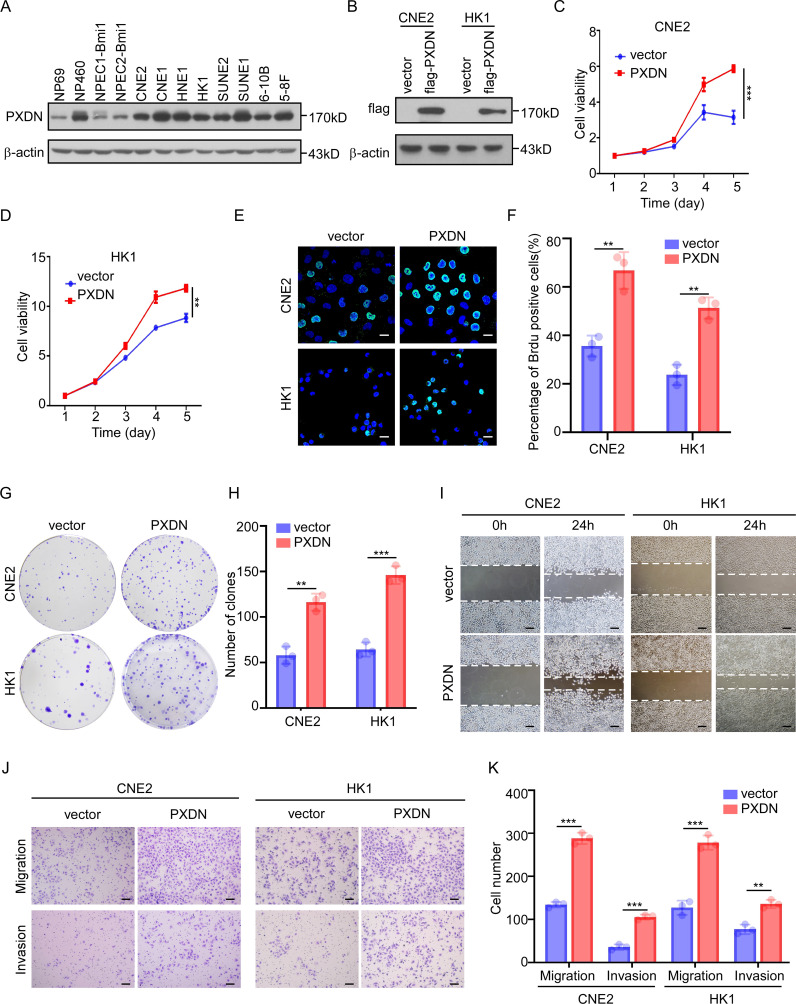
PXDN promotes cell proliferation and metastasis. **(A)** Western blotting showing PXDN expression in immortalized NPEC and NPC cell lines, β-actin is used as a loading control. **(B)** Western blotting showing the overexpression of PXDN in NPC cell lines. **(C, D)** CCK8 assay of vector or PXDN overexpression CNE2 **(C)** and HK1 **(D)** NPC cell lines, n = 4 biological replicates, *P* <0.0001 and = 0.0043 for CNE2 and HK1, respectively. **(E, F)** Brdu incorporation assay of vector and PXDN overexpressing CNE2 and HK1 NPC cell lines. Representative images are shown in **(E)**, the percentage of Brdu positive cells per field of view were plotted in **(F)**. The results are from three different experiments. Scale bars represent 20 µm. *P* = 0.0035 and 0.0014 for CNE2 and HK1, respectively **(G, H)** Colony formation of vector and PXDN overexpressing CNE2 and HK1 NPC cell lines. Representative images **(G)** and number of colonies **(H)** as shown. The results are from three different experiments. *P* = 0.0016 and 0.0003 for CNE2 and HK1, respectively**. (I)** wound healing assay of vector and PXDN overexpressing CNE2 and HK1 NPC cell lines. The results are from three different experiments. Scale bars represent 100 µm. **(J, K)** migration and invasion assays of vector and PXDN overexpressing CNE2 and HK1 NPC cell lines. Representative images are shown in **(J)**, the migrated and invaded cells per field of view were plotted in (**K**). The results are from three different experiments. *P* <0.0001 (migration) and *P* = 0.0002 (invasion) for CNE2, *P* = 0.0004 (migration), and *P* = 0.0022 (invasion) for HK1. Scale bars represent 100 µm. *P*-values were determined by unpaired t-test. **P <0.01, ***P <0.001.

To determine the biological function of PXDN, we measured PXDN protein levels in NPC cell lines. We found that PXDN expression was higher in most NPC cell lines than in the immortalized nasopharyngeal epithelial cells (**Figure 5A**). We then established two stable cell lines (CNE and HK1) with PXDN overexpression via retroviral infection (**Figure 5B**) and knockdown of endogenous PXDN expression in HNE1 and SUNE1 cell lines using specific siRNAs (**Supplementary Figure 1A**). CCK8 assays showed that overexpression of PXDN dramatically increased cell growth of both CNE2 and HK1 cells (**Figures 5C, D**). The BrdU incorporation assay also confirmed that overexpression of PXDN significantly increased NPC cell proliferation (**Figures 5E, F**). Consistently, ectopic expression of PXDN enhanced colony formation in NPC cells compared to that in control cells (**Figures 5G, H**). In addition, PXDN overexpression also promoted wound healing (**Figure 5I**), migration, and invasion (**Figures 5J, K**), consistent with the hypothesis that PXDN affects cell metastasis according to the GO analysis. In contrast, knockdown of PXDN in NPC cell lines resulted decreased proliferation (**Supplementary Figures 1B, C**), colony formation (**Supplementary Figures 1D, E**), wound healing ability (**Supplementary Figure 1F**), and metastatic ability (**Supplementary Figures 1G, H**). Previously, GO analysis suggested that PXDN might affect the ECM pathway. To assess the impact of PXDN on the ECM, we examined the protein levels of FN1, COL1A1, and COL3A1, which were increased at the mRNA level (**Figure 4C**). PXDN overexpression increased the protein expression of FN1, COL1A1, and COL3A1, suggesting the activation of ECM signaling by PXDN (**Supplementary Figure 1I**). Collectively, these results suggest that PXDN promotes the proliferation and metastasis of NPC cells, emphasizing the oncogenic role of PXDN in NPC.

### PXDN expression is negatively with T-cell infiltration in tumor microenvironment

**Figure 6:**
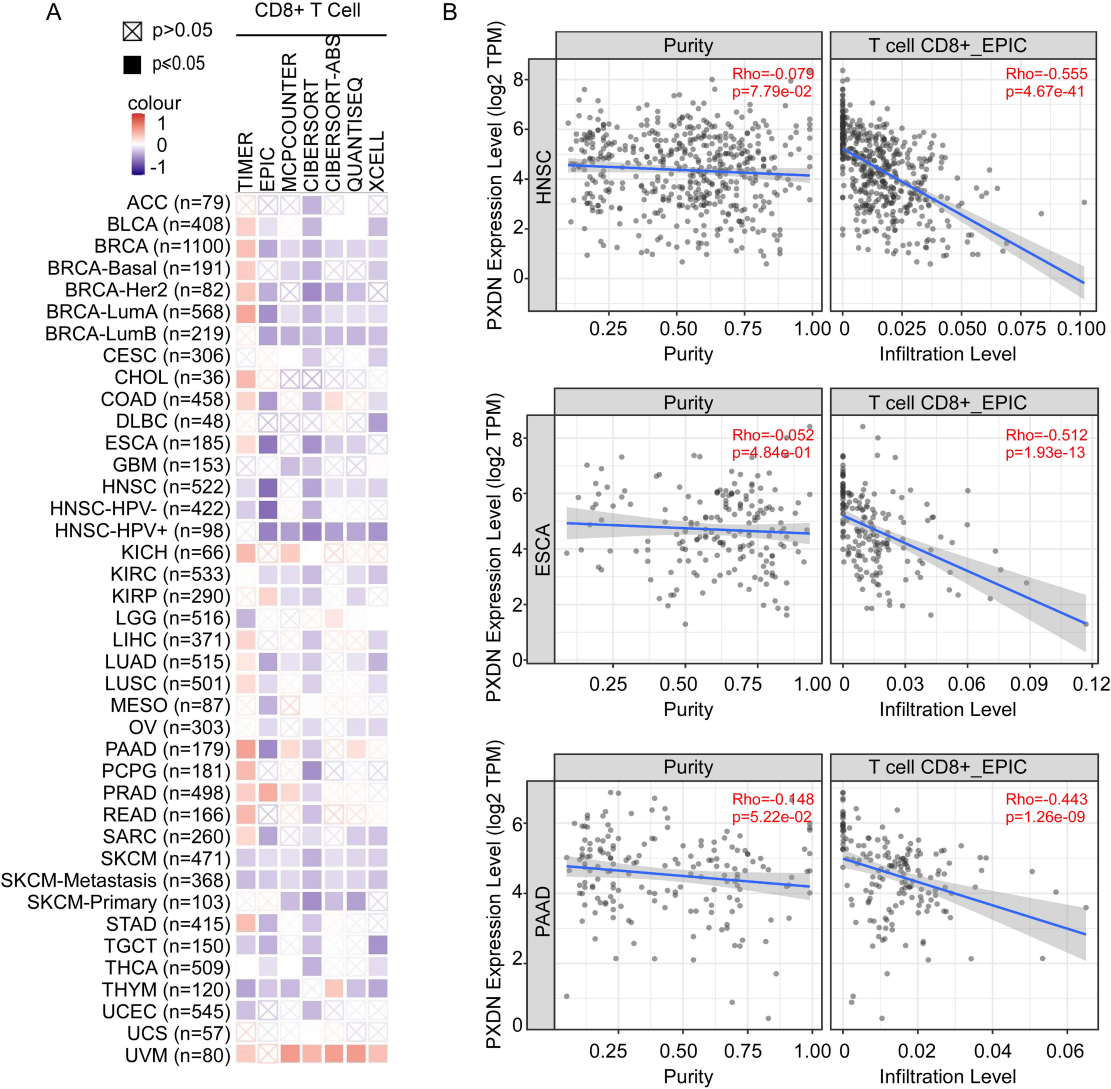
PXDN expression is negatively with T-cell infiltration in tumor microenvironment. **(A)** TIMER, EPIC, MCPCOUNTER, CIBERSORT, CIBERSORT-ABS, QUANTISEQ, and XCELL algorithms were used for the correlative analysis of the level of CD8+ T cell and the expression levels of PXDN across all tumors in TCGA. The red color indicates a positive correlation (0–1), the blue color represents a negative correlation (−1–0). The correlation with P-value <0.05 is considered as statistically significant. Statistically non-significant correlations values are marked with a cross. **(B)** PXDN expression is negatively correlated with the degree of infiltration of CD8+ T cells in HNSC, ESCA, and PAAD tissues.

The immune microenvironment is mainly composed of tumor cells, and their surrounding immune and inflammatory cells, tumor-associated fibroblasts, nearby interstitial tissues, microvessels, and various cytokines and chemokines. It is a complex integrated system that plays an important role in tumor development and immunotherapy. As shown above (**Figure 4**), GO analysis indicated that high PXDN expression decreased immune-associated pathways, such as leukocyte cell–cell adhesion, T-cell activation, and lymphocyte differentiation. To explore the relationship between PXDN expression and immune cell function in the tumor microenvironment, we used TIMER, EPIC, MCPCOUNTER, CIBERSORT, CIBERSORT-ABS, QUANTISEQ, and XCELL algorithms to comprehensively analyze the relationship between PXDN expression and the degree of T-cell infiltration in the tumor microenvironment. Interestingly, we discovered a significant negative correlation between PXDN expression and the estimated infiltration value of T cells in almost all tumor types (except melanoma) (**Figure 6A**), among which head and neck tumors, esophageal cancer, and pancreatic cancer were the most obvious (**Figure 6B**). Taken together, these data suggest that high expression of PXDN in tumors may cause T-cell dysfunction in the tumor microenvironment, leading to T-cell exhaustion, promoting tumor immune escape, and promoting tumor progression.

### High PXDN expression could potentially inhibits T-cell infiltration and proliferation in NPC

**Figure 7:**
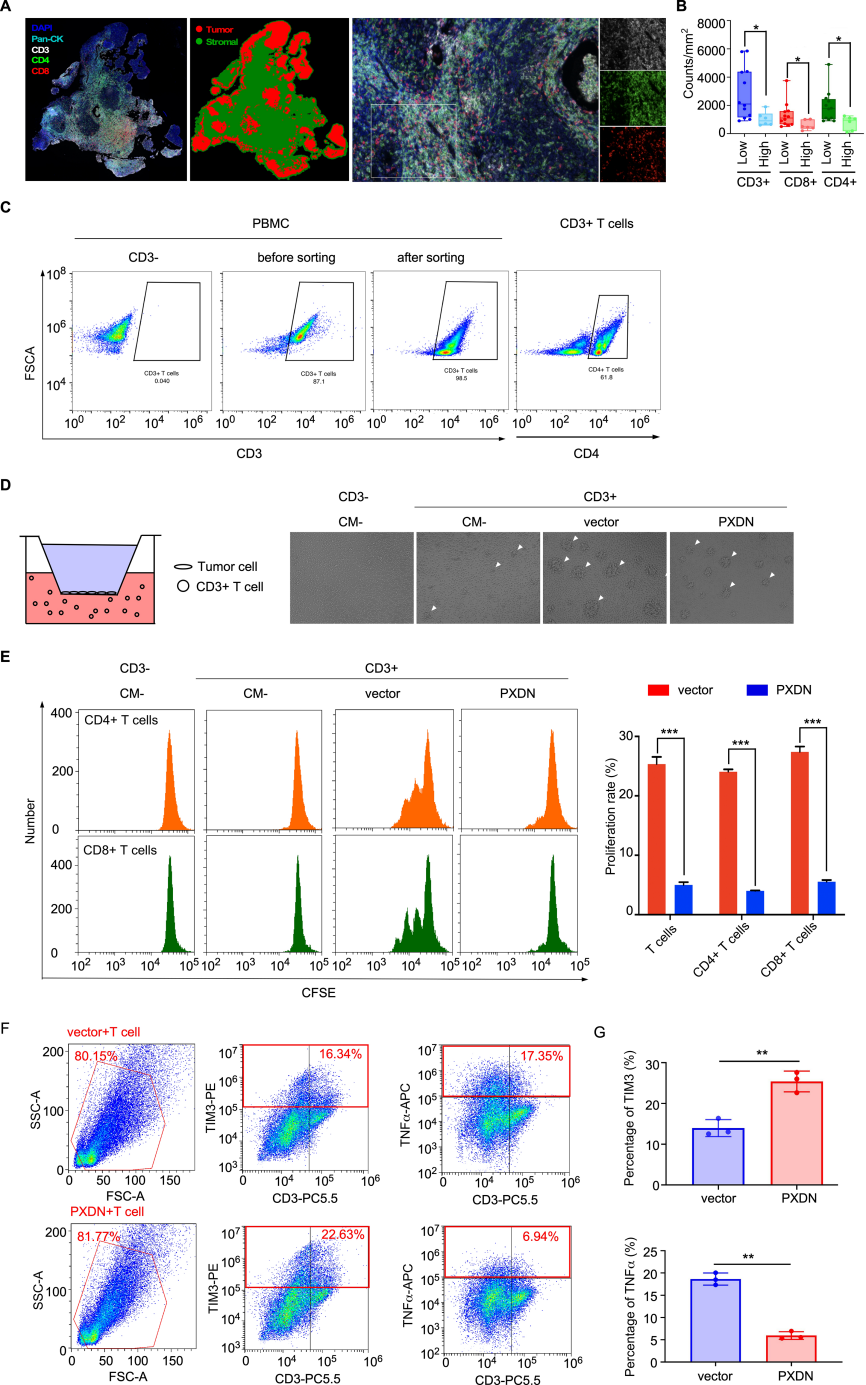
High PXDN expression inhibits T-cell infiltration and proliferation in NPC. **(A)** Multiple immunohistochemistry staining T cells (CD3, CD4, and CD8) and tumor cells (pan-CK) in NPC tissue. **(B)** The number of indicated T cells in NPC tissues with high (n = 12) or low (n = 6) expression of PXDN. *P* = 0.0143, 0.0229, and 0.0228 for CD3, CD4, and CD8, respectively **(C)** T cells were isolated from PBMC by specific magnetic beads. **(D)** Schematic diagram of co-culture of PXDN overexpressed NPC cells and T cells (left), proliferating balloon clone formed by T cells after co-culture (right). **(E)** Detection of T cell proliferation rate through CFSE flow cytometry. The results are from three different experiments. *P <*0.0001 for T cell, CD4 T cell, and CD8 T cell. **(F)** Cytotoxic T cell (TNFα) and suppressor T cell (TIM3) markers were detected after co-culture of PXDN overexpressed stable cell line and T cells. **(G)** Highly expression of PXDN promotes the proportion of inhibitory T cells (TIM3) and reduces cytokine secretion of tumor-killer T cells (TNFα). The results are from three different experiments. *P* = 0.002 and *P* = 0.0038 forTIM3 and TNFα, respectively. *P*-values were determined by unpaired t-test. *P <0.05, **P <0.01, ***P <0.001.

To further explore the effect of PXDN expression on T-cell infiltration in NPC tissue, multiple immunohistochemical (mIHC) staining was used to analyze the number of T cells in NPC tissue. CD3, CD4, and CD8 were used to identify T cells and pan-CK was used to identify the NPC cells (**Figure 7A**). The mIHC results showed that the number of T cells (CD3+) was significantly reduced in tissues with high PXDN expression, and the subpopulations of CD8+ T cells and CD4+ T cells were also significantly reduced (**Figure 7B**). Next, we isolated T cells from human blood mononuclear cells (PBMC) by specific magnetic beads (**Figure 7C**) and co-cultured the isolated T cells with PXDN overexpressed NPC cells. The growth of T cell clusters was significantly inhibited in T cells co-cultured with PXDN overexpressed NPC cells, suggesting that PXDN could potentially inhibit the growth of T cells (**Figure 7D**). The proliferation rate of T cells was detected by a CFSE-based flow cytometry assay, and the results showed that the proliferation rate of T cells was significantly reduced after co-culture with PXDN- overexpressing cell lines (**Figure 7E**). To further confirm whether PXDN affects T-cell function in the tumor microenvironment, after co-culture of T cells with PXDN-overexpressing NPC cell lines, cytotoxic T cell (TNFα) and suppressor T cell (TIM3) markers were detected by flow cytometry (**Figure 7F**). The results showed that the proportion of immunosuppressive T cells (TIM3+ T cells) was significantly increased and the tumor-killing factor TNFα secreted by T cells was significantly reduced, showing a state of T-cell exhaustion (**Figure 7G**). In conclusion, these results suggest that PXDN is highly expressed in tumor cells, which can potentially inhibit T-cell proliferation and infiltration in the microenvironment, which probably contributes to T-cell exhaustion and promotes the immune escape of NPC cells, ultimately promoting NPC progression.

## Discussion

The present study investigated the expression profile of PXDN in various cancer types and uncovered new roles for PXDN in promoting cancer progression. Our results demonstrate that elevated PXDN levels can distinguish cancer from normal individuals and predict poor survival in cancer patients. We further confirmed that PXDN was highly expressed in NPC and was associated with poor prognosis in NPC patients. Signal pathway analysis revealed that high expression of PXDN was associated with extracellular matrix activation and inhibition of immune cell activation. Functionally, the high expression of PXDN promotes NPC cell proliferation and metastasis. Furthermore, PXDN expression was negatively correlated with T-cell infiltration. Co-culture experiments confirmed that high PXDN expression potentially inhibited T-cell proliferation and anti-tumor immunity.

Currently, PXDN is reported to play a critical role in tissue development and homeostasis and is involved in cardiovascular disease, type 2 diabetes, and hepatocyte fibrosis (3–5). Several recent studies have identified an important link between PXDN and tumors such as oral squamous cell carcinoma, glioblastoma, and lung cancer (10, 23, 24). A systematic pan-cancer analysis provides a comprehensive understanding of the carcinogenicity of PXDN and suggests that it may be a potential target for tumor immunotherapy, providing a new candidate that could improve the clinical diagnosis and treatment of cancer (25). However, there are no relevant research reports on whether PXDN is involved in the occurrence and development of NPC. In this study, we conducted an in-depth analysis of PXDN expression in cancer using several databases, including TCGA, GTEx, CPTAC, and GEO. We conclude that PXDN could serve as a pan-cancer biomarker. We investigated the biological functions of PXDN in an NPC model. We performed transcriptome sequencing in NPC and found that high expression of PXDN was associated with ECM activation and immune inhibition. The ECM plays an important role in cancer metastasis and immune cell infiltration (26, 27). Molecular biology experiments validated the pro-tumor role of PXDN by promoting NPC cell proliferation and metastasis. Further research is needed to confirm the detailed mechanism involved in ECM in pro-tumor function and immune cell infiltration in NPC.

Immune cell infiltration plays an important role in cancer progression (28). NPC is a malignant tumor associated with EBV infection that exhibits a large amount of immune cell infiltration in the tumor microenvironment (29). However, the oncogenes that pronote NPC progression by affecting T-cell function in the tumor microenvironment need to be further explored. In this study, we found that the downregulated genes affected by PXDN were associated with immune cells, especially T cells, adhesion, and activation. Immune analysis revealed a negative correlation between PXDN expression and CD8+ T-cell infiltration. Furthermore, we explored T-cell infiltration in NPC tissues and found that NPC tissues with high PXDN expression showed lower T-cell infiltration of both CD8+ and CD4+ T cells. We co-cultured T cells with PXDN overexpressed NPC cells and found that PXDN overexpression inhibited T-cell proliferation and anti-tumor activation, as demonstrated by the decreased levels of the cytotoxic T cell marker (TNFα) and the increased expression of the suppressor T cell marker (TIM3) marker. These results suggest that PXDN may induce T-cell exhaustion in the tumor microenvironment and promote the immune escape of NPC cells to alter NPC progression.

In conclusion, we have characterized PXDN as a valuable biomarker for pan-cancer diagnosis and prognosis. The mutation features of PXDN are also correlated with clinical survival in different cancers, especially UCEC. Biological function investigation confirmed the oncogenic role of PXDN by increasing NPC cell proliferation and metastasis. Mechanistically, PXDN promotes ECM signaling and potentially suppresses T-cell infiltration and activation, thereby facilitating cancer progression. Nevertheless, the current study was not able to elucidate the *in vivo* function and detailed mechanism by which PXDN acts as an oncogene, which will be conducted in our future research.

## Conclusion

PXDN is a valuable biomarker for the diagnosis and prognosis of cancer. We also uncovered new oncogenic roles for PXDN in promoting cancer progression and regulating T-cell immunosuppressive function.

## Data Availability

The original contributions presented in the study are included in the article/**Supplementary Material**. Further inquiries can be directed to the corresponding authors.
