# Supplementary material for: PXDN as a pan-cancer biomarker and promotes tumor progress via immune inhibition in nasopharyngeal carcinoma
Source: Front Oncol. 2024 Sep 27;14:1463011. doi: 10.3389/fonc.2024.1463011 (PMC11466884; doi:10.3389/fonc.2024.1463011)
Supplement: Supplementary file 1 [file DataSheet1.pdf]

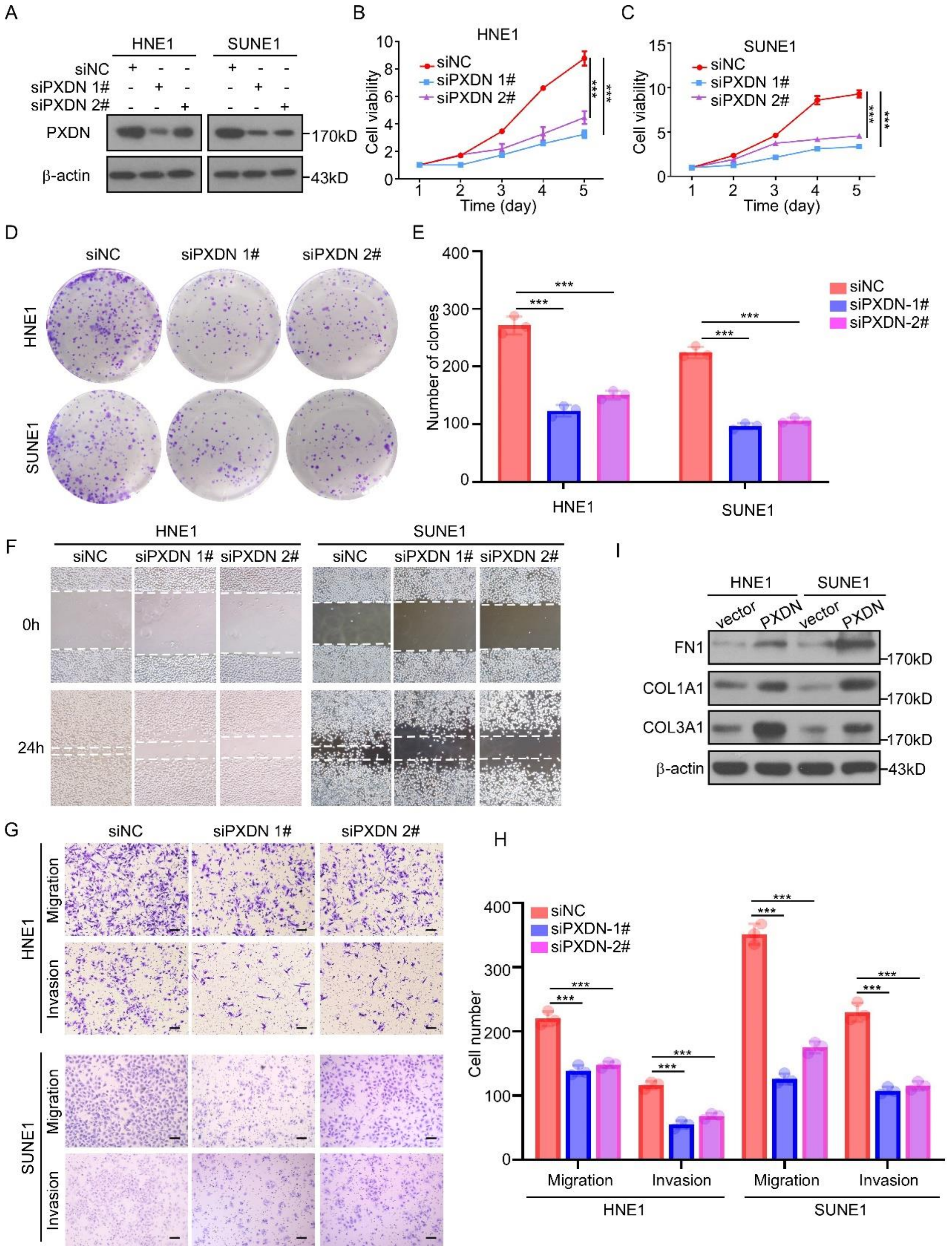

Supplementary Figure 1. Knockdown of PXDN decreases cell proliferation and metastasis

A. Western blotting showing the knockdown of PXDN in HNE1 and SUNE1 NPC cell lines. **B-C**. CCK8 assay of siNC or si-PXDN in HNE1 **(B)** and SUNE1 **(C)** NPC cell lines, n=4 biological replicates. **D-E**, Colony formation of siNC or si-PXDN in HNE1 and SUNE1 NPC cell lines. Representative images (**D**) and number of colonies (**E**) as shown. The results are

from three different experiments. **F,** wound healing assay of siNC or si-PXDN in HNE1 and SUNE1 NPC cell lines. The results are from three different experiments. Scale bars represent 100 µm. **G-H**, migration and invasion assays of siNC or si-PXDN in HNE1 and SUNE1 NPC cell lines. Representative images are shown in (**G**), the migrated and invaded cells per field of view were plotted in (**H**). The results are from three different experiments. Scale bars represent 100 µm. **I,** Western blotting showing the ECM signal in PXDN overexpressed CNE2 and HK1 cell lines. *P*-values were determined by unpaired t-test. $^*P < 0.05$, $^{**}P < 0.01$, $^{***}P < 0.001$.

Supplementary Table1: Characteristics of the patients.

Characteristics	No.of patients	Expression of PXDN		P value[a]
		Low	High	
Patients	57	29	28	
Age				
Median	46			
Range	27~67			
≤47	28	12	16	0.294
＞47	29	17	12	
Gender				
Female	13	3	10	**0.029**
Male	44	26	18	
BMI				
＞24	22	12	10	0.787
⩽24	35	17	18	
T classification				
T1-T2	6	1	5	0.102
T3-T4	51	28	23	
N classification				
N0-N1	26	16	10	0.186
N2-N3	31	13	18	
TNM				
III	37	18	19	0.783
IV	20	11	9	
VCA-IgA				
＜1:160	28	16	12	0.431
≥1:160	29	13	16	
EA-IgA				
＜1:20	32	15	17	0.596
≥1:20	25	14	11	
EBVDNA				
⩽4000	27	16	11	0.292
＞4000	30	13	17	
Progression				
Yes	18	5	13	**0.024**
No	39	24	15	
Death				
Yes	13	3	10	**0.029**
No	44	26	18	

[a]Chi-square test

Supplementary Table 2. Univariate and Multivariate analysis with the COX proportional hazards for the predictor of OS and DFS of the NPC patients

Prognostic factors	Univariate analysis				Multivariate analysis			
	p	HR	95% CI for HR		*p*	HR	95% CI for HR	
			Lower	Upper			Lower	Upper
OS								
Age	0.313	0.561	0.183	1.722				
Gender	0.079	0.366	0.119	1.125				
BMI	0.774	1.174	0.392	3.516				
T stage	0.580	0.652	0.143	2.969				
N stage	0.119	2.792	0.767	10.158				
TNM stage	0.127	2.348	0.784	7.028				
VCA-IgA	0.077	3.232	0.882	11.842				
EA-IgA	0.978	0.985	0.324	2.994				
EBV DNA	**0.026**	5.529	1.223	24.995	**0.047**	4.659	1.019	21.306
PXDN	**0.021**	4.584	1.258	16.709	**0.043**	3.829	1.042	14.071
DFS								
Age	0.529	0.742	0.293	1.881				
Gender	0.094	0.431	0.161	1.153				
BMI	0.721	0.841	0.326	2.173				
T stage	0.114	0.367	0.106	1.271				
N stage	0.204	1.886	0.708	5.028				
TNM stage	0.630	1.165	0.626	2.170				
VCA-IgA	0.160	2.021	0.757	5.393				
EA-IgA	0.624	1.260	0.500	3.178				
EBV DNA	**0.019**	3.776	1.241	11.492	**0.040**	3.238	1.057	9.925
PXDN	**0.014**	3.659	1.301	10.287	**0.030**	3.154	1.114	8.926

Age (years), (>46 vs.≤46); Gender, female vs male; BMI, (>24 vs.≤24); T stage, T3-T4 vs T1-T2; N stage, N2-N3 vs N0-N1; TNM stage, IV vs III, VCA-IgA, ≥1:160 vs <1:160; EA-IgA, ≥1:20 vs <1:20; EBV DNA(copy/ml), >4000 vs ≤4000; PXDN, high vs ＜low.
